# Performance Analysis of MIMO-STBC Systems with Higher Coding Rate Using Adaptive Semiblind Channel Estimation Scheme

**DOI:** 10.1155/2014/304901

**Published:** 2014-02-12

**Authors:** Ravi Kumar, Rajiv Saxena

**Affiliations:** Department of Electronics & Communication Engineering, Jaypee University of Engineering & Technology, A.B. Road, Raghogarh, Guna, Madhya Pradesh 473226, India

## Abstract

Semiblind channel estimation method provides the best trade-off in terms of bandwidth overhead, computational complexity and latency. The result after using multiple input multiple output (MIMO) systems shows higher data rate and longer transmit range without any requirement for additional bandwidth or transmit power. This paper presents the detailed analysis of diversity coding techniques using MIMO antenna systems. Different space time block codes (STBCs) schemes have been explored and analyzed with the proposed higher code rate. STBCs with higher code rates have been simulated for different modulation schemes using MATLAB environment and the simulated results have been compared in the semiblind environment which shows the improvement even in highly correlated antenna arrays and is found very close to the condition when channel state information (CSI) is known to the channel.

## 1. Introduction

The need for speed, reliability, and quality in the wireless digital communication has lured the interest of vast community of technologist and communication forums to have a look at the multiple-input multiple-output (MIMO) technology that has proved a vital role in satisfying their needs [1]. Nowadays wireless networks are utilizing many techniques such as wireless local area network (WLAN), wireless sensor networks (WSN), and personal area network (PAN) which demands more spectrum and makes it costlier and precious resource. This technology has comprehensive uses like in worldwide interoperability for Microwave Access, providing a wireless alternative to cable and the digital subscriber line (DSL) for the leased line last mile access without compromising the speed as the MIMO provides high data rate. This has attracted the attention of the researchers towards the use of multiple antennas at both transmitter and receiver which has been known as MIMO antenna array systems. MIMO nowadays has been widely used for enhancing the channel capacity using multiple antenna configurations in both 3G and 4G technologies adopting the influence over the wireless local area network (WLAN) as the IEEE 802.11 *n* standard which has reached the acquired level as high as 600 Mbps [2]. The advantages are not only bound to the communication field but they are also injected in the medical field in sensing of the cardiopulmonary activity [3]. In MIMO systems, the data rate may be enhanced by using spatial multiplexing whereas the reliability may be enhanced by using space time coding (STC). Space time block codes (STBCs) have been implemented in MIMO systems for increasing the diversity gain or coding gain by coding across multiple antennas over multiple symbol durations. Initially, STBC was analyzed by [4], which further was modified by [5] using maximum likelihood (ML) technique. Each antenna element on a MIMO system operates on the same frequency and therefore requires no extra bandwidth and it also requires less power than that required by a single antenna. Advantage of MIMO systems has been taken as Beamforming, spatial diversity and spatial multiplexing [6, 7].

Transmit diversity has been studied extensively for combating the fading channels because of its simple implementation using multiple antenna at the transmitter end. The first bandwidth efficient transmit diversity scheme was proposed by [8] with the delay diversity scheme proposed by [9] which further was modified by [10] giving multilayered space time architecture. Alamouti introduced the well-known STBC in [11] for two transmit and one receive antennas. STBC consists of data coded with the space and time to improve the reliability of the transmission. Later, [12, 13] introduced orthogonal space time block coding which generalizes Alamouti's transmission scheme to an arbitrary number of transmit antennas and is able to achieve the full diversity promised by the multiple transmit and receive antennas. Like Alamouti scheme, these generalized codes have a very simple maximum likelihood decoding algorithm based only on linear processing at the receiver. Space time block coding generalizes the transmission and arbitrary number of transmit antennas and is able to achieve full diversity with respect transmit and receive antenna. It is a set of practical signal design techniques to which approaches the information theoretic capacity limits of MIMO systems. In last few years, some of the techniques have been evolved like orthogonal space time block coding [5, 14], quasiorthogonal space time block coding (QOSTBC) [15, 16], and nonorthogonal STBC (NOSTBC) [17]. Generally all the MIMO-STBC systems require space time equalizer at the receiver end for decoding purpose which also requires the channel state information (CSI) which is basically obtained through training based techniques at the expense of bandwidth efficiency. These training based techniques are also in research for utilizing minimum bandwidth and increasing robustness to detect the signal with minimum number of transmitted training symbols for which the semiblind techniques are being utilized. Pilot assisted semiblind technique has also evolved in [18], which has been successfully applied to MIMO systems for obtaining remarkable enhancement. The performance comparison among various designs of pilot assignments using different kinds of modulation schemes and interpolation techniques for frequency offset estimation was proposed by [19, 20]. Some of the techniques do not require CSI [21–24] and pay the penalty in performance of at least 3dB as compared to coherent maximum-likelihood (ML) receivers. The drawback of completely blind channel estimation is the inability to detect the signals appropriately from the channel to the receiver. The desired signal can be easily identified and extracted with the help of some training sequences. The aim of identifying the signal is to recognize the signal with strength with as much less training symbols as possible, for which the semiblind channel estimation technique has become the prominent technique these days. Blind channel estimation algorithms based on ML technique have been proposed by [25, 26]. Iterative methods have been utilized to avoid the computational complexity of the ML detection technique, the cyclic ML [26, 27], and the expectation maximization (EM) [27, 28] algorithms. These iterative methods require a careful initialization of the channel or symbol in case otherwise poor initializing can strongly affect the symbol-error rate (SER). However, excluding some specific low rate codes, different approaches fail to extract the channels in a full blind manner whereas these can be implemented using the semiblind channel estimation technique. Several approaches have been suggested in the literature to solve this problem, including the transmission of the short training sequence [29, 30] or the use of precoders [31–33]. However these semiblind techniques cannot be employed in a noncooperative scenario since they need to modify the transmitter, which has been implemented utilizing the adaptive pilot assisted channel estimation scheme (APACE) proposed by [18] and by some modification at the precoder and decoder in the proposed scheme, based on which the analysis has been shown in [34] for the STBC with code rate up to 1. Despite the literature available universally, none of the algorithms is able to estimate the channel matrix for general STBCs without modification of the pilot training symbols and precoding. In this paper, a new approach has been proposed to implement the MIMO-STBC system with the implementation of the proposed adaptive pilot assisted semiblind channel estimation (APASBCE) scheme [18]and modifying the code rate to some higher levels. This paper describes the brief extract on MIMO systems, with respect to channel capacity, system model, and channel models including the focus on spatial diversity, that is, STBC.

## 2. Space Time Block Coding (STBC)

### 2.1. STBC for Real Constellations

Considering *M*_*T*_ × *M*_*R*_ transmission matrix with variables *s*_1_, *s*_2_,…, *s*_*ns*_ satisfying [35, 36]

(1) SMT·SMTT=c|S1|2+|S2|2+⋯+|Sns|2IMT,

where *c* is a constant and *I*_*M*_*T*__ is a *M*_*T*_ × *M*_*R*_ identity matrix, STBC can achieve a full diversity of order of 1. Square STBC matrix *S*_*M*_*T*__ with real constellation can be found if and only if the number of transmit antennas is *M*_*T*_ = 2,4, or 8. These codes offer full transmit diversity of *M*_*T*_ due to their full rate *R* = 1. The real transmission matrices for 2, 4, and 8 transmit antennas are given by

(2) S2=[s1s2−s2s1],  S4=[s1s2s3s4−s2s1−s4s3−s3s4s1−s2−s4−s3s2s1],S8=[s1s2s3s4s5s6s7s8−s2s1s4−s3s6−s5−s8s7−s3−s4s1s2s7s8−s5−s6−s4s3−s2s1s8−s7s6−s5−s5−s6−s7−s8s1s2s3s4−s6s5−s8s7−s2s1−s4s3−s7s8s5−s6−s3s4s1−s2−s8−s7s6s5−s4−s3s2s1].

At the receiver end, the received expressions are based on Alamouti's model with the simplicity of having only real symbols and therefore no conjugate symbol in the equations. Thus the received expressions for any number of received antennas become

(3) r1,NR=rNRt=h1,NRs1+h2,NRs2+n1,NR,r2,NR=rNRt+T=−h1,NRs2+h2,NRs1+n2,NR,

where *n*_1,*N*_*R*__, *n*_2,*N*_*R*__ are independent noise samples, *h*_*i*,*N*_*R*__ is the channel transfer function from the *i*th transmit antenna and *N*_*R*_ denotes the *M*_*T*_ receive antenna. Received signals are then combined for two transmit antennas as

(4) s~1=∑NR=1MRr1,NRh1,NR+r2,NRh2,NR,s~2=∑NR=1MRr1,NRh2,NR−r2,NRh1,NR.

Similarly, the received signal for four and eight transmit antennas can also be derived. Alamouti STBC do not require CSI at the transmitter and can be used with two transmit antennas and 1 receive antenna with accomplishment of full diversity of 2. It reduces the effect of fading at receiver station at the cost of some additional antenna elements at the transmitter end. If having more antennas is not a problem, then this scheme is appropriate for getting full diversity of 2*N*_*R*_ with two transmit antennas.

### 2.2. STBC for Complex Constellation

For STBC with complex constellation, if *M*_*T*_ × *M*_*R*_ transmission matrix with variables *s*_1_, *s*_2_ …, *s*_*ns*_ satisfies [35, 36]

(5) SMT·SMTT=c|s0|2+|s1|2+⋯+|sns|2IMT,

where *c* is a constant and *I*_*M*_*T*__ is a *M*_*T*_ × *M*_*R*_ identity matrix, STBC can achieve full diversity of the order of 1. The full rank diversity that was introduced by Alamouti is considered the simplest STBC with complex constellation and it is also the only *M*_*T*_ × *M*_*R*_ STBC code with complex constellation, which is the only STBC achieving full rate of 1 for a full diversity of 2. For the case of 3 transmit antennas, [4] made block codes with 1/2 and 3/4 code rates and full diversity 3 *M*_*R*_. The aim of using higher number of transmit antennas on generalized STBC is to achieve high rate with full diversity, minimum coding delay *n*_*s*_, and minimum decoding complexity. Examples of half rate complex transmission matrices achieving full diversity for three and four transmit antennas are given as

(6) S3C=[s1s2s3−s2s1−s4−s3s4s1−s4−s3s2s1∗s2∗s3∗−s2∗s1∗−s4∗−s3∗s4∗s1∗−s4∗−s3∗s2∗],  S4C=[s1s2s3s4−s2s1−s4s3−s3s4s1−s2−s4−s3s2s1−s1∗s2∗s3∗s4∗−s2∗s1∗−s4∗s3∗−s3∗s4∗s1∗−s2∗−s4∗−s3∗s2∗s1∗],

where (*) denotes the complex conjugate of the element. The matrix *S*_3_^*C*^ code transmits 4 symbols every 8 time intervals and therefore has rate 1/2. For both schemes, flat fading channel are assumed to be constant over 8 symbol periods. Thus the received constellation derivations and the received signals can be formed as in (4) and then combined to retrieve the original transmitted symbols using maximum likelihood detection to minimize the decision metric which can also be formed for four transmit antenna. If three transmit antennas are considered and, three symbols are transmitted every four time intervals, and therefore has code rate 3/4. Example of 3/4 code rate complex transmission matrix for three transmit antennas is given as

(7) S3C=[s1s2s32−s2∗s3∗s32s3∗2s3∗2(−s1−s1∗+s2−s2∗)2s3∗2−s3∗2(s2+s2∗+s1−s1∗)2].

It is known that the complexity at the receiver end increases linearly with the number of transmit antennas and the receive antennas. Indeed, for *X* receiving antennas, the expression of matrix will have *X* times more terms than that it has now. Performance of STBC for complex constellation matrices of 1 bit/s/Hz, 2 bits/s/Hz, and 4 bits/s/Hz for two transmit antennas and 1/2 bit/s/Hz, 1 bit/s/Hz, and 2 bit/s/Hz for three and four transmit antennas has already been analyzed.

### 2.3. Orthogonal Space Time Block Codes

As shown earlier, examples of 1/2 and 3/4 code rate complex transmission matrices for four transmit antennas have been proposed by [36] which gave full diversity of 4*N*_*R*_. With four transmit antennas and code rate of 1/2 and 3/4, complex transmission matrices have been given as

(8) S4C(12)=[s1s2s3s4−s2s1−s2s1−s3s4s1−s2−s4−s3s2s1s1∗s2∗s3∗s4∗−s2∗s1∗−s4∗s3∗−s3∗s4∗s1∗−s2∗−s4∗−s3∗s2∗s1∗],S4C(34) =[s1s2s32s32−s2∗s1∗s32−s32s3∗2s3∗2−s1−s1∗+s2−s2∗2−s2−s2∗+s1−s1∗2s3∗2−s3∗2s2+s2∗+s1−s1∗2−s1+s1∗+s2−s2∗2].

### 2.4. Quasi-Orthogonal Space Time Block Codes

Full rate STBCs, using complex symbols in their transmission matrix, are not possible to achieve as we have seen in previous section. Indeed, the particular case of Alamouti code presented can only achieve full rate with full diversity which follows the rules of orthogonal design for simple decoding. The new STBC technique called quasiorthogonal STBC (QOSTBC) is proposed by [15], which achieved full rate at the cost of higher complexity decoding. Quasiorthogonal designs are attractive because of their achievement of higher code-rate than orthogonal designs and lower decoding complexity than nonorthogonal designs. As suggested in [15],

(9) S4Q=[s2cs1,s2s2cs3,s4−s2c∗s3,s4s2c∗s1,s2]=[s1s2s3s4−s2∗s1∗−s4∗s3∗−s3∗−s4∗s1∗s2∗s4−s3−s2s1].

Now, *V*_*i*_, *i* = 1,2, 3,4, is defined as the *i*th column of *A*; it is easy to see that (*V*_1_, *V*_2_) = (*V*_1_, *V*_3_) = (*V*_2_, *V*_4_) = (*V*_3_, *V*_4_) = 0, where (*V*_*i*_, *V*_*j*_) = ∑_*l*=1_^4^(*V*_*i*_)_*l*_(*V*_*j*_)_l_* is the inner product of vectors *V*_*i*_ and *V*_*j*_. Therefore, the subspace created by *V*_1_ and *V*_4_ is orthogonal to the subspace created by *V*_2_ and *V*_3_. This orthogonality allows the calculation of the maximum likelihood decision metric. Indeed the maximum likelihood detection is to find the pair (*s*_1_, *s*_4_) and (*s*_2_, *s*_3_) that minimizes *f*_14_(*s*_1_, *s*_4_) over all possible values of (*s*_1_, *s*_4_) and minimizes *f*_23_(*s*_2_, *s*_3_) over all the possible values of (*s*_2_, *s*_3_) pairs. It seems that the complexity of the decoder increases as compared to the STBC decoder presented earlier. However, complexity of QOSTBC does not grow linearly as for STBC but exponentially with the number of transmit and receive antennas. Similarly, the QOSTBC code with different rate and higher number of transmit antennas has also been proposed.

## 3. System Model

**Figure 1:**
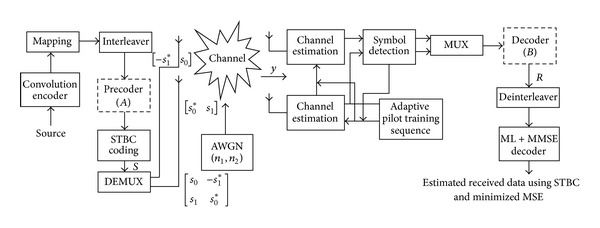
System block model using Alamouti's STBC method.

Consider an *N*_*T*_ × *N*_*R*_ quasistatic Rayleigh flat fading MIMO channel, where *N*_*T*_ and *N*_*R*_ denote the number of transmit and receive antennas. The system is described by *y*(*k*) = *Hx*(*k*) + *n*(*k*), where *x* is [*x*_1_(*k*),*x*_2_(*k*),…,*x*_*M*_*T*__(*k*)]^*T*^ which is the transmitted symbol vector of *M*_*T*_ transmitter, *y* denotes the received vector *y*(*k*) = [*y*_1_(*k*),*y*_2_(*k*),…,*y*_*M*_*R*__(*k*)]^*T*^, and *n*(*k*) = [*n*_1_(*k*),*n*_2_(*k*),…,*n*_*M*_*R*__(*k*)]^*T*^ is the complex valued Gaussian white noise vector at the receiving end for MIMO channels with energy *E*[*n*(*k*)*n* ^*H*^(*k*)] = 2*σ*_*n*_^2^ *I*_*M*_*R*__ distributed according to *N*_*c*_(0, *σ*_*n*_^2^ *I*_*M*_*R*__) assumed to be zero mean, white, and independent of both channel and data fades. The channel model considered here is denoted by *H* = *R*_*R*_^1/2^ *H*_*ω*_ *R*_*T*_^1/2^ [37] with *R*_*T*_ and *R*_*R*_ representing the normalized transmit and receive correlation matrices with identity matrix. The entries of *H*_*ω*_ are independent and identically distributed (*i*.*i*.*d*.) *N*_*c*_ (0, 1). A system block diagram using Alamouti's method is shown in Figure 1. The transmitting symbols are encoded according to orthogonal STBC scheme. A pilot sequence is inserted in the transmission of every **X** symbol which will be reduced with the implementation of the proposed adaptive semiblind estimation scheme in [18]. A different pilot scheme has been used for each channel and these orthogonal pilot sequences enable the receiver to decouple pilot sequences from the combined signals for each channel at a receive antenna. The transmitted symbols have been considered having empty slots left in their codeword matrix for maintaining the orthogonality between the symbols of the vector.

Assuming the block transmission scheme with block length *T*, the *n*th received data block can be expressed as

(10) y(k)=D(k,ω0)x(k)+n(k),

where

(11) Y(K)=[y1{(k−1)T+1}y2{(k−1)T+2}⋮y(kT)],X(K)=[x1{(k−1)T+1}x2{(k−1)T+2}⋮x(kT)],n(K)=[n1{(k−1)T+1}n2{(k−1)T+2}⋮n(kT)].

Here, *D*(*k*, *ω*_0_) is defined as

(12) D(k,ω0)=diag⁡{ejω0(k−1)T+1⋯ejω0kT},

where the (·)^*T*^ denotes the transpose in the second exponential term. If a slow fading environment is considered, the time becomes much longer than the data block length *T*. The matrix *X*(*k*) = *X*{*s*(*k*)} can be treated as a mapping transforming the *k*th block to *T* × *K* complex matrix of transmit signals, where *s*(*k*) = {*s* ^(1)^ *s* ^(2)^ ⋯ *s* ^(*L*)^}^*T*^ is the *k*th symbol vector alphabet set of length *L*, that is, set of all possible symbol vectors. The *T* × *K* matrix *Xs*(*k*) is called an OSTBC if all elements of this matrix are linear functions of the *K* complex variables *s*_1_(*k*), *s*_2_(*k*),…, *s*_*n*_(*k*) and their complex conjugates. The calculation of the basis function of OSTBC can be denoted by

(13) X(s(k))=∑k=1K[CkRe{sk(n)}+Ck+KIm⁡{sk(n)}],

where

(14) $$\begin{array}{c} C_{k}=\{\begin{array}{cc} X\left(e_{k}\right), & \text{for}\;\;1\leq k\leq K\\ X\left(je_{k-K}\right), & \text{for}\;\;K+1\leq k\leq 2K, \end{array} \end{array}$$

where *Re*{·} and *Im*⁡{·} denote the real and imaginary parts. It is known that OSTBC is completely defined by its basis matrices {*C*_*k*_}_*k*=1_^2*K*^. If the channel frequency offset is not available, then (10) can be rewritten in vectorized form and the 2*M*_*T*_ *T* × 2*K* real valued matrix can be denoted by, *X*(*H*) = [*C*_1_ *H* *C*_2_ *H* ⋯ *C*_2*K*_ *H*]. The *X*(*H*) matrix follows the decoupling property; that is, its columns have identical norms and are orthogonal to each other.

(15) X(H)XT(H)=||H||F2I2K,

where ||·||_*F*_ denotes the Frobenius norm of a matrix. *X*(*k*, *S*(*k*)) follows the basis matrices, and is referred to as a time varying OSTBC [34].

## 4. Design Condition and Decoding Method

It has been found that *Xs*(*k*) denotes an OSTBC for *M*_*T*_ transmit antennas which transmit *k* information symbols *x*_1_(*k*), *x*_2_(*k*),…*x*_*M*_*T*__(*k*) with having empty slots left in its codeword matrix for orthogonality; we then found *k* + *λ* information symbols transmitting high code rate with full diversity *X*_*n*,*k*+*λ*_ from *Xs*(*k*) as

(16) Xn,k+λ=Xs(k)+PWλ,

where *W*_*λ*_ is the codeword matrix with *λ* additional information symbols to be transmitted from empty slots of *Xs*(*k*) and *P* is the optimization matrix-wise entries that are with both *Xs*(*k*) and *PW*_*λ*_ being nonoverlapping entries. Owing to the nonorthogonal structure of the information symbols, as unknown deterministic parameters, it is required to apply ML estimation approach to jointly estimate both the symbols and pilots. To obtain the ML estimates of all these parameters, the log-likelihood function needs to be maximized. Hence the parameter estimates can be found by solving the following optimization problem:

(17) max⁡H max⁡slog⁡f{y(1),y(2),…y(n) ∣ S,h},

where *f*{*y*(1), *y*(2),…*y*(*n*) | *S*, *h*} is the likelihood function computed for *N* snapshots *y*(*n*)_*n*=1_^*N*^ and *s* is the set of all possible values of the transmitted symbols received. It is not easy to solve (17) because its computational cost grows exponentially in *N*. To simplify the optimization problem in (17), we have to maximize the expectations and minimize the error in the estimates for which

(18) X^=arg min⁡x1,x2,…xk+λ||Y−Xn,k+λH||2.

Now, the elimination of terms coming from additional transmitted symbols from empty slots of *Xs*(*k*) will be tried by computing intermediate signals from the received signals for all possible values of the additional symbols *x*_*k*+1_, *x*_*k*+2_,…*x*_*k*+*λ*_ in *W*_*λ*_ as

(19) $$\begin{array}{c} Z=Y-PW_{\lambda }H \end{array}$$

and the optimization problem in (17) can be rewritten as

(20) max⁡h,Slog⁡f{y(1),y(2),…y(n) ∣ S,h}.

The likelihood function for any *y*(*n*) can be expressed as

(21) f{y(n) ∣ H}=(1(πσ2)MTT)(−||y(n)−X(n,h)H(n)||2/2),

where *E*{·} denotes the statistical expectation. Taking into account that all {*y*(*n*)}_*n*=1_^*N*^ are independent random vectors, the obtained value is given as

(22) $$\begin{array}{c} f\left\{y\left(1\right),y\left(2\right),\ldots y\left(n\right)\;|\;s,h\right\}=\overset{N}{\underset{n=1}{\prod }}f\left\{y\left(n\right)\;|\;H\left(n\right),h\right\}. \end{array}$$

Using (21) and (22), the problem in (20), can be formulated as shown in (18) and can also be written as

(23) min⁡h,S∑n=1N||y(n)−X(n,h)H(n)||2.

It is known in (23) that the *n*th term of the sum is minimized with

(24) H(n)=1||h||2XT(n,h)y(n),

where (24) follows from the fact that *X*(*n*, *h*) satisfies the decoupling property that has been discussed earlier in (15). Using this equation, the objective function in (23) can be concentrated with respect to {*H*(*n*)}_*n*=1_^*N*^ and, after such concentration, the latter optimization problem can be shown as

(25) min⁡h∑n=1N||y(n)−X(n,h)XT(n,h)y(n)||h||2||2.

This function can further be solved in a simple manner and found with the existence of traces of matrix as

(26) ∑n=1N||y(n)−X(n,h)XT(n,h)y(n)||h||2||2 =1||h||2∑n=1Ntr⁡{X(n,h)XT(n,h)y(n)yT(n)}+constant.

Now, with the little replacements in the expression for convenience, equation can be denoted by

(27) =vec⁡T{X(n,h)}(I2K⊗y(n)yT(n))vec⁡{X(n,h)}=hTΦT(n)(I2K⊗y(n)yT(n))Φ(n)h,

where Φ(*n*) is 4*KM*_*T*_ *T* × 2*M*_*T*_ *N* matrix whose *k*th column can be defined as [Φ(*n*)]_*k*_ = *vec*⁡{*X*(*n*), *e*_*k*_}, where *e*_*k*_ is the *k*th coloumn of the identity matrix *I*_2*MN*_ and ⊗ is the Kronecker matrix product. Now, putting (27) in (26), the concentrated optimization problem can be denoted by

(28) max⁡hhTΨ(ω0)h||h||2,

where

(29) Ψ(ω)=∑n=1NΦT(n)Φ(n)(I2K⊗y(n)yT(n)).

The above expression (29) is 2*M*_*T*_ *N* × 2*M*_*T*_ *N* real matrix which depends on the received data vectors {*y*(*n*)}_*n*=1_^*N*^ and the carrier frequency offset *ω*. Further, this can be solved and the carrier frequency offset can be derived using (28):

(30) ω^=arg max⁡ωλmax⁡{Ψ(ω)},

where Ψ(*ω*) denotes the largest eigenvalues of matrix. And further for the estimates of channel one has

(31) h^=P{Ψ(ω^)},

where *P*(·) denotes the normalized principal eigenvector of a matrix with the assumption of no multiplicity in the largest eigenvalues of Ψ(*ω*). Now for those specific OSTBCs that result in $\Psi \left(\hat{\omega }\right)$ with multiple largest eigenvalues, h belongs to the subspace spanned by the corresponding multiple principal eigenvectors of $\Psi \left(\hat{\omega }\right)$, and, as a result, the blind technique is not applicable using this method of detection. Hence, the semiblind technique proposed in [18] will be utilized which uses the small number of training symbols both in time and frequency axis adaptively and decoded at the receiver end according to the requirement of the channel as shown in [34]. Using this method, it searches for all the possible combinations of *x*_*k*+1_, *x*_*k*+2_,…*x*_*k*+*λ*_ and we use the decoding procedure of *Xs*(*k*) that is used to obtain conditional estimates to get the weight vectors in (18) of [18]. An adaptive method of increasing pilot symbols in the empty slots has been proposed and implemented in the same and then the robust estimation method has been found for getting the correct combination of *x*_*k*+1_, *x*_*k*+2_,…*x*_*k*+*λ*_. Finally, we minimize the decision metric in (18) for *x*_1_, *x*_2_,…*x*_*k*_, *x*_*k*+1_, *x*_*k*+2_,…*x*_*k*+*λ*_ is minimized over all possible values of *x*_*k*+1_, *x*_*k*+2_,…*x*_*k*+*λ*_. This method of estimating and detecting is somewhat similar to ML detection technique and therefore the total decoding complexity of *kM*_*T*_ × *M* ^*λ*^ = *kM*_*T*_^*λ*+1^ is obtained. Now, it is known that *h* belongs to the subspace spanned by {*u*_*l*_}_*l*=1_^*n*^,

(32) h=∑l=1nαlul=Uα,

where **U** = [*u*_1_, *u*_2_,…*u*_*n*_] and **α** = [*α*_1_, *α*_2_,…*α*_*n*_]^*T*^. The proposed semiblind channel estimation scheme has been utilized to obtain the estimate of **U** in a blind way and meanwhile estimating the vector **α** using the training symbols as low as possible. It is known that the number of entries in **α** is much less than that in **h**, and this semiblind estimator will require very less training data than the direct training based channel estimator obtaining all entries of **h** in a nonblind way. For this purpose, it is required to estimate the value of *α* and take short time average of the detected estimates and then further process it to give the branch metric which then further will proceed for giving the selected estimates with minimum branch metric which gives the minimum surviving states with minimum value from the $\hat{H}$ and eventually the possible block of transmitted sequence. The ML estimate for the STBC system of the vector **α** can be written as

(33) a^=(hr−h^)(hr−h^)T.

This further will give

(34) a^=(XXT)−1XTr,

where *r* = *Xα* + *n*. This estimate can be used to obtain the coefficients {*α*}_*l*=1_^*n*^ from these few training symbols to resolve the ambiguity in the channel vector estimate. To ensure that the ML estimate in (34) is unique, it is required that 2*M*_*T*_ *T* ≥ *n* and, for known nonidentifiable OSTBCs, *n* = 4 holds true and therefore, as *T* ≥ 2, the condition 2*M*_*T*_ *T* ≥ *n* is satisfied for any number of receive antennas for which it is required to have code rate of STBC that should be higher than 1 which will be further designed in the next section [34].

## 5. High Code Rate Design Method

In order to achieve energy efficient STBC codes with high code rates, it is required to construct the *M*′*X*[*i*]^2^, a rotated version of the complex lattice with source information *X*[*i*]^2^, where *M*′ is a complex unitary matrix, so that there is no shaping loss in the signal constellation emitted by the transmitting antenna as shown in [38].

For any given *M*_*T*_ and *G* column groups of the matrix and *T* being the block length, then *T* = *M*_*T*_ + 2(*G* − 1). Assuming that *M* is even, the higher code rate STBC will be designed as

(35) ΘMT,T,G=AMT,T,G′+jBMT,T,G′,

where the real and imaginary matrices *A*_*M*_*T*_,*T*,*G*_′ and *B*_*M*_*T*_,*T*,*G*_′ of size *T* × *M*_*T*_ are given as

(36) AMT,T,G′=[sR1sR2−sR2sR1],  BMT,T,G′=[sI1sI2sI2−sI1],

where *s*_*R*_^*i*^ and *s*_*I*_^*i*^ are real and imaginary parts of *S* ^*i*^, where *i* = 1,2, that is given as

(37) Si=sRi+jsIi =[sx′+10⋯0sx′+MT/2+1sx′+2⋱⋮⋮sx′+MT/2+2⋱0sx′+(G−1)(MT/2)+1⋮⋱sx′+MT/20sx′+(G−1)(MT/2)+2⋱sx′+MT⋮⋮⋱⋮0⋮⋱sx′+G(MT/2)],

where *x*′ = (*i* − 1)*G*(*M*_*T*_/2) and the *G*th diagonal layer from left to right written as (*M*_*T*_/2) × 1 vector *X*_*G*_^′*i*^  (*i* = 1,2;  *G* = 1,2,…*G*) is given as

(38) XG′i=[sx′+(G−1)(MT/2)+1⋯sx′+(G−1)(MT/2)+MT/2]T.

The symbol rate of the STBC code Θ_*M*_*T*_,*T*,*G*_ is given as

(39) Code  rate=LT=MTGMT+2G−2,

which is the same as that of STBC decoding proposed in [39, 40]. For a large value of *G*, the code rate can be up to *M*/2 and similarly for large elements on transmitting side, that is, *M*_*T*_, the code rate can be up to *G*. For the design of STBC with odd antenna elements, it is supposed to design an STBC for *M*_*T*_ + 1 transmit antennas with the last antenna to be shut down; that is, when the *M*_*T*_ is odd, the STBC is obtained by selection of first *M*_*T*_ columns of the STBC designed for *M*_*T*_ + 1 antennas.

## 6. Proposed Code Designs 

### 6.1. For Three Antenna Elements

In this section, new STBC code with code rates of 1.5 and 2 has been achieved for three transmit antennas with the use of the design procedure shown in the previous section. According to (16), using the design method as shown in previous section, for the transmitting six symbols using three antennas, that is, code rate 2 can be found using the value of *G* = 2 as

(40) A3,6,4′=[s1s2s5−s2∗s1∗−s6s7s8s3−s8∗s7∗−s4∗],  B3,6,4′=[s3s4s7−s4∗s3∗−s8∗s5s6s1−s6∗s5∗−s2∗]

with the optimization matrix,

(41) G=[11ejθ11ejθejθejθ1ejθejθ1],

where *A*_3,6,4_′ and *B*_3,6,4_′ denote the real and imaginary matrices for the 6 symbols per 3 transmit antennae with code rate 2. This matrix has been derived from the optimization matrix of *Ge* ^*jθ*^ *I*_4_, where *I*_4_ is the 4 × 4 identity matrix. After continuous simulation search, maximum coding rate of Θ_3,6,4_ has been found by sacrifice of some constellation angle *θ* for the optimum value of 65.49°, which gave minimum determinant value of 0.15 for the QPSK modulation technique.

Now, secondly, the real and imaginary matrices for code rate 1.5 which transmits six information symbols per three time intervals are obtained as

(42) A3,6,3′=[s1s2s5−s2∗s1∗−s600s300−s4∗],  B3,6,3′=[s3s40−s4∗s3∗−s6s5s6s1−s6∗s5∗−s2∗],

with the optimization matrix

(43) G=[11ejθ11ejθejθejθ1ejθejθ1].

Maximum coding rate of Θ_3,6,3_ has been found by making constellation angle *θ* for the optimum value of 44.96°, which gave minimum determinant value of 0.32 for the QPSK modulation technique. Hence in this, it can be easily seen that the complexity has been reduced upto 4*M*_*T*_^3^ for *M*_*T*_ = 4 and for *λ* = 2.

### 6.2. For Four Antenna Elements

In this section, another STBC code with higher code rates of 1.3, 1.5, and 2 has been achieved for four transmit antennas with the use of the design procedure shown in the earlier section. According to (16), using the design method as shown in previous section, for the transmitting six symbols using four antennas, that is, code rate 1.3 can be found using the value of *G* = 2 as

(44) A4,6,2′=[s10s50s3s2s7s60s40s8−s50s10−s7−s6s3s20−s80s4],B4,6,2′=[s10s50s3s2s7s60s40s8s50−s10s7s6−s3−s20s80−s4].

Maximum coding rate of Θ_4,6,2_ has been found by making constellation angle *θ* for the optimum value of 1.04°, which gave minimum determinant value with the QAM modulation technique.

With the QPSK modulation technique, for code rate 2, we found that the following matrix is formed as

(45) A4,8,4′=[s1s2s5s6−s2∗s1∗−s6∗−s5∗s7s8s3s3−s8∗s7∗−s4∗s3∗],B4,8,4′=[s3s4s7s8−s4∗s3∗−s8∗s7∗s5s6s1s2−s6∗s5∗−s2∗s1∗]

with optimization matrix given as

(46) G=[11ejθejθ11ejθejθ11111111].

Maximum coding rate was found for the code rate of 2, with the angle *θ* = 14.2° of QPSK signal constellation with symbols on the two-axis rotation to achieve full diversity.

Similarly, for the code rate 1.5 with the four antennas, using QPSK modulation rotation at *θ* = 90° can be obtained by making *s*_7_ = *s*_8_ = 0 which resulted as

(47) A4,8,3′=[s1s2s5s6−s2∗s1∗−s6s500s3s400−s4∗s3∗],B4,8,3′=[s3s400−s4∗s3∗00s5s6s1s2−s6∗s5∗−s2∗s1∗],

with the optimization matrix

(48) G=[11ejθejθ11ejθejθ11111111].

### 6.3. Decoding and Estimation Method

A decoding method for four antenna elements is being described here, in which the receiver calculates the intercepted received signals from the channel using (19) for all the combinations of *s*_*k*+1_, *s*_*k*+2_,…*s*_*k*+*λ*_ to obtain the ML estimates of *s*_1_, *s*_2_,…*s*_*k*_. Therefore for the given values of *s*_5_, *s*_6_, *s*_7_, and *s*_8_ which can only be obtained with the help of reduced form of (19),

(49) Z=Θ4,8,4H+N.

It is required to minimize the decision metric obtained with the help of (18) for all possible values and obtained conditional ML estimates of *s*_1_, *s*_2_, *s*_*k*_ which need additional decoding complexity of *kM*_*T*_ per each step of *M*_*T*_^*λ*^ calculations. Therefore we get a total decoding complexity of *kM*_*T*_ × *M*_*T*_^*λ*^ = *kM*_*T*_^*λ*+1^. The receiver follows the decoding procedure of Θ_4,8,4_, and it is observed that *Z*_*ij*_ which is component of *Z* is calculated from *y*_*ij*_ which is component of *Y*; here *i* and *j* are the column and row of the corresponding matrix. The receiver combines the received intercepted signals to obtain

(50) y^1=∑i=1MRhi,1∗Zi,1+hi,2Zi,1∗;  y^2=∑i=1MRhi,2∗Zi,1+hi,1Zi,2∗;y^3=∑i=1MRhi,3∗Zi,3+hi,4Zi,4∗;  y^3=∑i=1MRhi,4∗Zi,3+hi,3Zi,4∗.

These received estimates can now be utilized for the estimation of ML estimates for *s*_*i*_, where *i* = 1,2, 4 manipulated for estimating higher code rate estimates:

(51) s1ML=argmin⁡s1⁡[(β′|s^1A′−α′s1A′|2)+(α′|s^1B′−β′s1B′|2)],s2ML=argmin⁡s2⁡[(β′|s^2A′−α′s2A′|2)+(α′|s^2B′−β′s2B′|2)],s3ML=argmin⁡s3⁡[(β′|s^3A′−α′s3A′|2)+(α′|s^3B′−β′s3B′|2)],s4ML=argmin⁡s4⁡[(β′|s^4A′−α′s4A′|2)+(α′|s^4B′−β′s4B′|2)],

where *A*′ and *B*′ denote the real and imaginary parts of STBC codes *α*′∑_*i*=1_^*M*_*R*_^(|*h*_*i*,1_|^2^, |*h*_*i*,2_|^2^), β′=∑i=1MR(|hi,3|2+|hi,4|2)s^1=Re(y^1)+jIm⁡(y^3), s^2=Re(y^3)+jIm⁡(y^1), and s^3=Re(y^4)+jIm⁡(y^2).

It is observed that a total decoding complexity of 4*M*_*T*_^5^ rather than *M*_*T*_^8^ by minimizing (18) for all the possible values as discussed earlier has been achieved. 

Now, for the implementation of the estimated symbols with the semiblind channel estimation, it is required to deploy the scheme proposed in [41], although it can also be implemented with [18], but the procedure to estimate has been refined in the second method by modifying precoder and decoder at the transmitter and receiver side implementing the same estimation technique used in [18]. It can be seen in (29) of [41] that the weight vectors are not sufficient to estimate the symbols correctly in the semiblind environment with partial CSI conditions and an adaptive estimation method was tried as shown in [18] for getting optimal linear minimum mean square error (MMSE) estimate for the channel path gain at the *m*th symbols period where the weighting coefficients *h*(*i*, *q*) explicitly depend on the symbol position. For each *q*, *h*(*i*, *q*) can be obtained by solving the adaptive method as discussed in [18] which provides the unknown estimate sequence *α* to avoid sacrifice of tracking ability of channel. These estimated symbols can be used to obtain the coefficients {*α*_*l*_}_*l*=1_^*N*_*R*_^ from these few training symbols to resolve the ambiguity in the channel vector estimate. The minimum path metric with its short time average of long detected sequence *μ*_*k*_ was detected which has then been utilized to calculate the minimum branch metric *μ*_*k*_ for all possible estimated vectors for tracking surviving state with minimum value of channel coefficients $\hat{H}$. Then these metrics are utilized to update the weight vectors in (29) of [41] of *m*th spatial equalizer at each step with increase in processing steps *k*. Reference [41] has discussed the capacity analysis of the proposed semiblind channel estimation scheme with modified precoder and decoder. In this paper, Bit Error Rate performance analysis has also been taken care of. A comparative chart has been shown in Table 1 for showing the used number of antennas for different schemes and their symbol transmission rate with different coding rates.

## 7. Results Analysis and Conclusion

Performance analysis and improvement observed in the MIMO systems using different antenna configurations utilizing STBC using different modulation schemes with the implementation of Adaptive pilot assisted semiblind channel estimation scheme for the partial CSI condition proposed earlier in [18] have been shown in this section. It is known that the performance for the different STBC coding schemes degrades when more bits per symbol are transmitted, but we have simulated up to 9 bps/Hz with higher code rate STBCs which has shown relatively good results. For the general simulation case for known channel models, it is obtained that the best performance is obtained by using higher number of transmitting and receiving antenna elements. However, for any modulation case with low SNR values, three-ransmitting-antenna STBC system with code rate 1/2 gives better results than the four-antenna-element system STBC with code rate 3/4 even though the gain for the said is higher. When the simulation was tried with more numbers of antenna elements at the transmitting side with code rate 1/2, they gave better results than the 3/4 code rate type STBC systems. The possible reason for this is that the higher rate of four-transmitting-antenna element system causes lower channel gain per symbol and therefore BER for particular SNR. If we consider equal data rates, and simulate the 16-QAM scheme and 64-QAM modulation scheme for the code rate that is, 1/2 and 3/4, for three and four antenna element systems, it is easily observable that the *S*_3_^*C*^and *S*_4_^*C*^ with code rate 3/4 using 16-QAM (4 bits/symbol) gave the same data rate, as given by *S*_3_^*C*^and *S*_4_^*C*^ with code rate 1/2 using 64-QAM (6 bits/symbols). Hence we decided to show the comparative analysis of QPSK and 16-QAM modulation schemes for different antenna configurations with maintaining the correlation coefficient of 0.5.

In this section, we have evaluated the BER performance and the received constellation comparisons for different modulation schemes using STBC with different code rates, for their constellation angle maintaining the appropriate modulation for achieving the exact code rate and diversity. Also the capacity comparison is shown for the improvement seen with different antenna configurations with different channels. Throughout the simulations, the noise variance has been considered between the 3 dB and 20 dB level for different scenarios.

**Figure 2:**
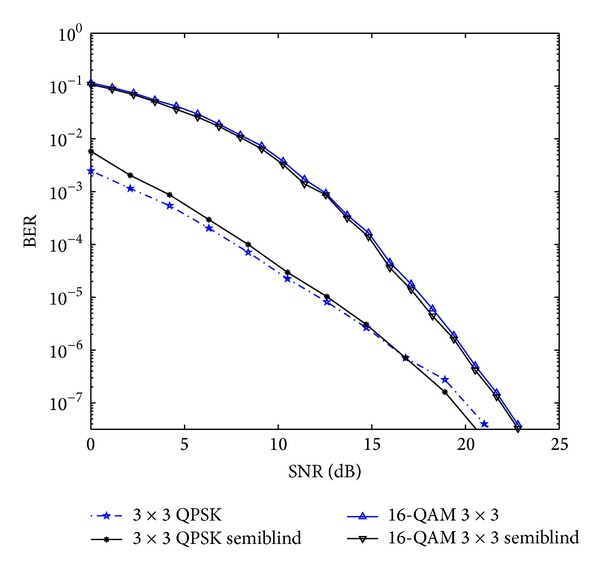
Comparative results of Alamouti's model using QPSK and 16-QAM modulation techniques for 3 × 3 transreceivers antenna with APASBCE based simulation using the STBC found in (6) with code rate 1/2 and diversity 1.

**Figure 3:**
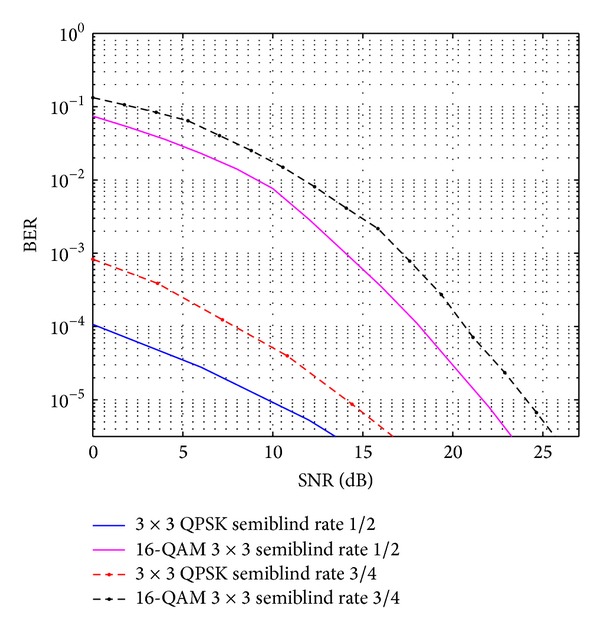
OSTBC comparison for 3 × 3 antenna systems with the APASBCE based simulation using QPSK and 16-QAM modulation techniques for code rates 1/2 and 3/4 using *S*_3_^*C*^ in (6) and (7).

**Figure 4:**
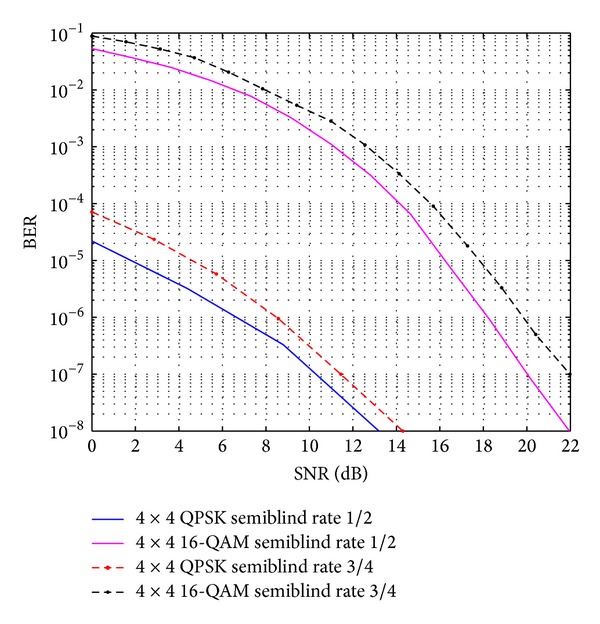
OSTBC comparison for 4 × 4 antenna systems with the APASBCE based simulation using QPSK and 16-QAM modulation techniques for code rates 1/2 and 3/4 using *S*_4_^*C*^ in (6) and (8).

**Figure 5:**
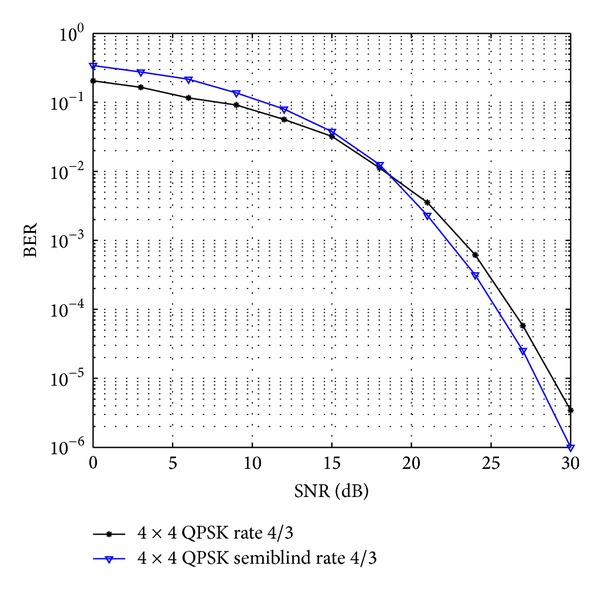
OSTBC comparison for QPSK modulation for 4 × 4 antenna systems with the APASBCE based simulation using the STBC found in (44) with code rate 1.3 and diversity 1.

The comparative result analysis has been shown in Table 2 for different STBCs with different transmitting antenna configurations with their respective code rates. The APASBCE scheme has been implemented with the Alamouti's model using QPSK and 16-QAM modulation technique for 3 × 3 antenna configuration using STBC found in (6) with code rate 1 and diversity order 1. In Figure 2, the improvement for 3 × 3 antenna configuration with QPSK has been observed after the 17.4 dB SNR level and 12 dB SNR level for 16-QAM. It is seen that the semiblind result gave better result than the existing results available in the literature as the number of iterations reaches up to the level when the symbols are easily identifiable at the receiver end. Figures 3 and 4 show that code rate 1/2 is performing better than code rate 3/4 for both 3 × 3 and 4 × 4 antenna configurations, as discussed earlier in this section, but Figure 4 shows significant improvement in the BER between the simulated semiblind results as compared with Figure 3. This happens because of the increase in number of antenna elements; as the number of elements increases, the symbol rate increases, and further by utilizing the proper code rate STBC, the BER may be enhanced upto some extent which has been shown in Figure 4. Similarly, Figure 5 is showing the comparison of OSTBC for QPSK modulation for 4 × 4 antenna configuration with proposed estimation scheme with the block codes found in (44) with code rate 1 and diversity 1 in which the improvement has been observed after 18.8 dB SNR level.

**Figure 6:**
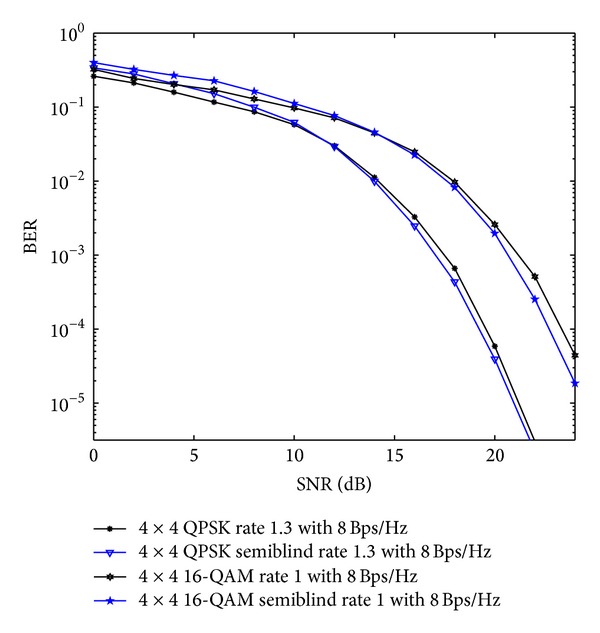
QOSTBC and OSTBC comparison for 4 × 4 antenna systems with the APASBCE based simulation using QPSK and 16-QAM modulation techniques for code rates 1 and 1.3 using *S*_4_^*C*^ in (9) and *A*′ and *B*′ in (44).

**Figure 7:**
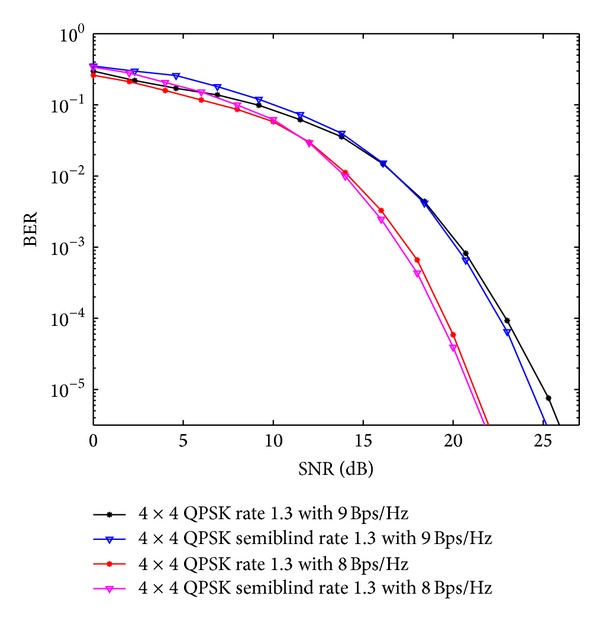
OSTBC comparison for 4 × 4 antenna systems with the APASBCE based simulation using QPSK with the existing results for code rate 1.3 with 8 Bps/Hz and 9 Bps/Hz using *A*′ and *B*′ in (44).

**Figure 8:**
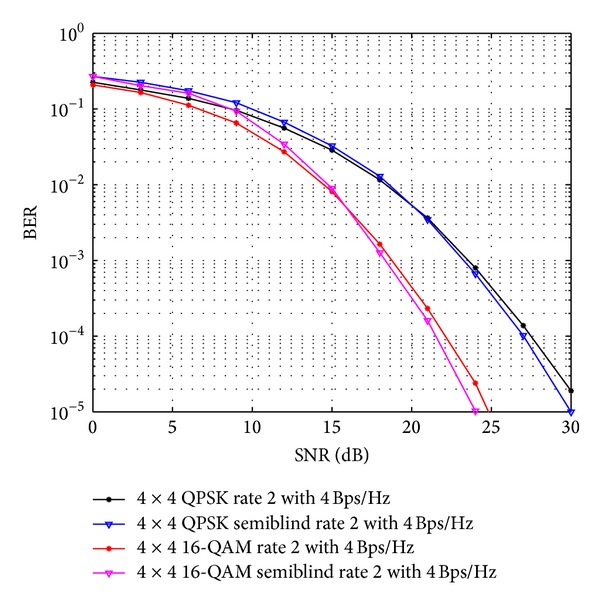
OSTBC comparison of 4 × 4 antenna systems with the existing result and APASBCE based simulation results using QPSK and 16-QAM modulation technique for code rate 2 with 4Bps/Hz using *A*′ and *B*′ in (45).

**Figure 9:**
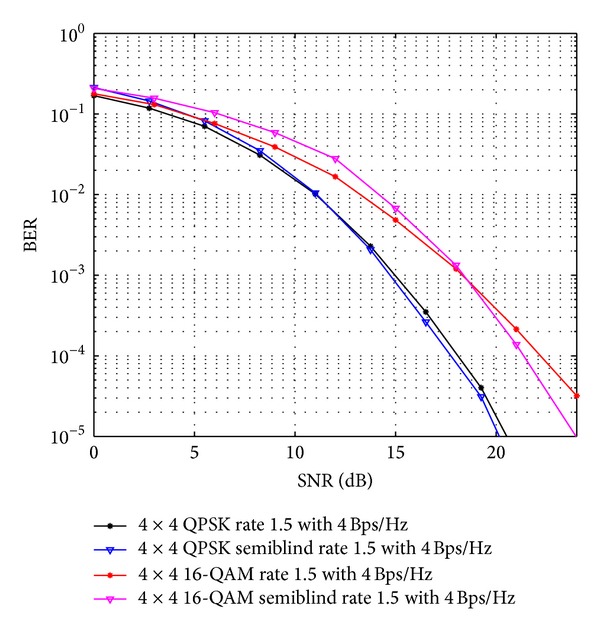
OSTBC comparison of 4 × 4 antenna systems with the existing result and APASBCE based simulation results using QPSK and 16-QAM modulation technique for code rate 1.5 with 3 Bps/Hz using *A*′ and *B*′ in (47).

In Figure 6, comparisons for QOSTBC and OSTBC using 4 × 4 antenna configuration with proposed scheme has been done using *S*_4_^*C*^ in (9) and *A*′ and *B*′ in (44) using QPSK and 16-QAM modulation scheme for code rates 1 and 1.3, respectively, with same bit rate of 8 Bps/Hz. The improvements in this figure has been observed after 14.2 dB SNR level for 16-QAM and after 11.4 dB for QPSK modulation scheme but before reaching the BER level of 10^−2^ which is the advantage in this category. Similarly, Figure 7 is showing the improvement of OSTBC comparison for 4 × 4 antenna configuration using QPSK modulation with APASBCE scheme After 11.8 dB SNR level for 8 Bps/Hz bit rate and after 16.7 dB for 9 Bps/Hz bit rate before reaching the BER level of 10^−2^ with the existing results for code rate 1.3 using block codes found in *A*′ and *B*′ of (44). As discussed earlier, the higher rate of transmitting antenna element system causes lower channel gain per symbol and therefore BER for particular SNR value it is seen in this figure that low rate, that is, 8 Bps/Hz, for QPSK modulation is performing better than the higher rate, that is, 9 Bps/Hz. It is observed in Figure 8, for QPSK modulation, that the system is able to maintain 1.2 dB gain at the level of 25 dB of SNR, for the STBC with code rate 2, whereas in case of 1.5 code rate, STBC simulations, as depicted in Figure 9, were able to maintain less amount of gain nearly of 0.8 dB but at the SNR level of 20 dB. Secondly, for 16-QAM modulation, as evident in Figures 8 and 9, the effect of maintaining gain is not of the same quantum, and maintains the gains of 2.8 dB and 2 dB for APASBCE based STBC with code rates 2 and 1.5, respectively, at the higher level of SNR that is, 25 dB, and 30 dB. The decrease in maintaining less SNR gain has occurred because of the loss of training symbols at the receiver end for which the algorithm again started to track the symbols, and after finding the sufficient amount of training symbols, it kept maintaining the gain in SNR levels again.

**Figure 10:**
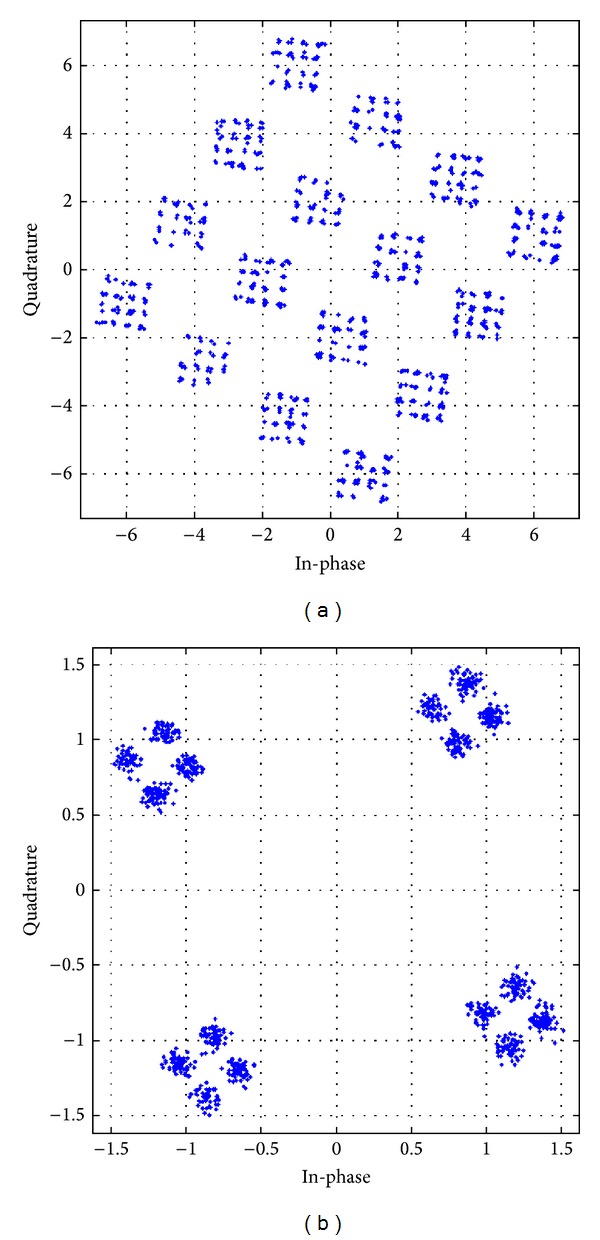
Received constellation for Alamouti's 3 × 3 antenna system with code rate 1 with discriminating levels at SNR 23 dB for 16-QAM modulation with angle 36.89° in (a) and QPSK modulation with angle 8.92° in (b).

**Figure 11:**
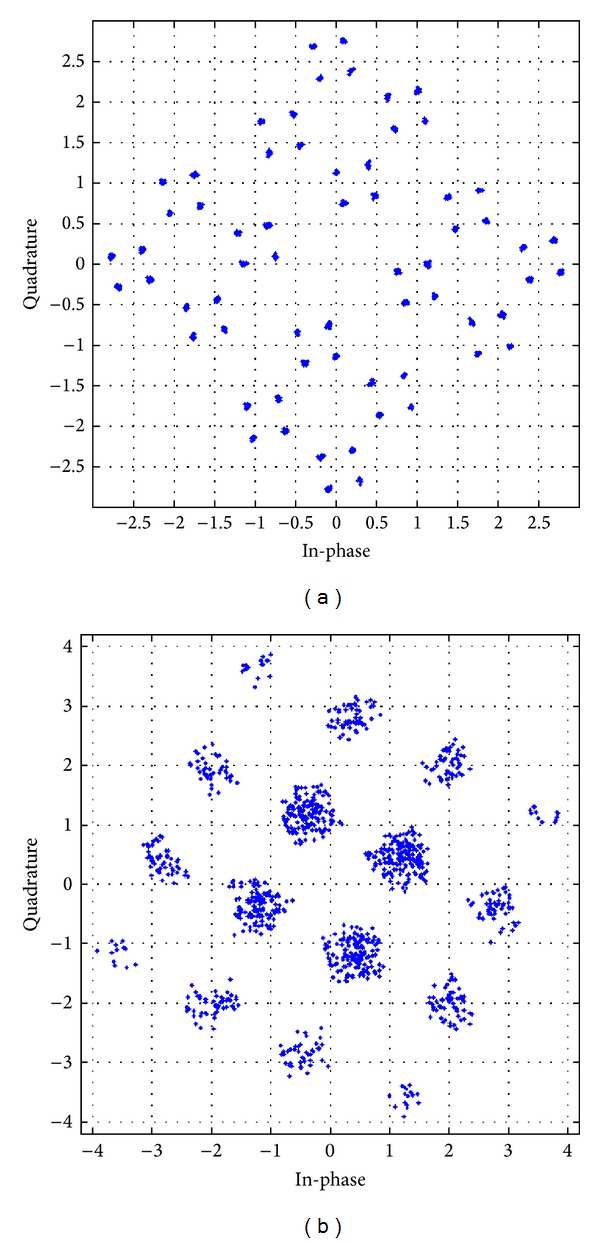
Received constellation for OSTBC MIMO antenna systems with code rate 1/2 with discriminating levels at SNR 35 dB and 25 dB for 3 × 3 and 4 × 4 antenna systems in (a) and (b), respectively, using QPSK modulation technique with angle 1.5° in (a) and 18.48° in (b).

**Figure 12:**
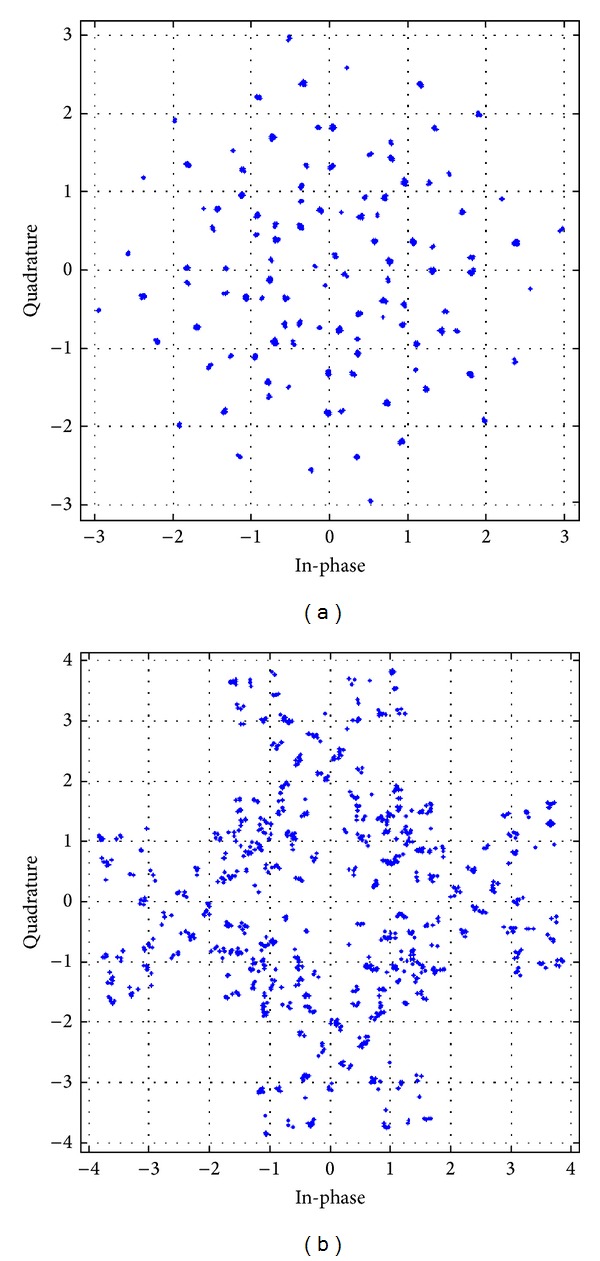
Received constellation for OSTBC MIMO antenna systems with code rate 3/4 with discriminating levels at SNR 40 dB and 30 dB for 3 × 3 and 4 × 4 antenna systems in (a) and (b), respectively, using QPSK modulation technique with angle 1.8° in (a) and 3.2° in (b).

**Figure 13:**
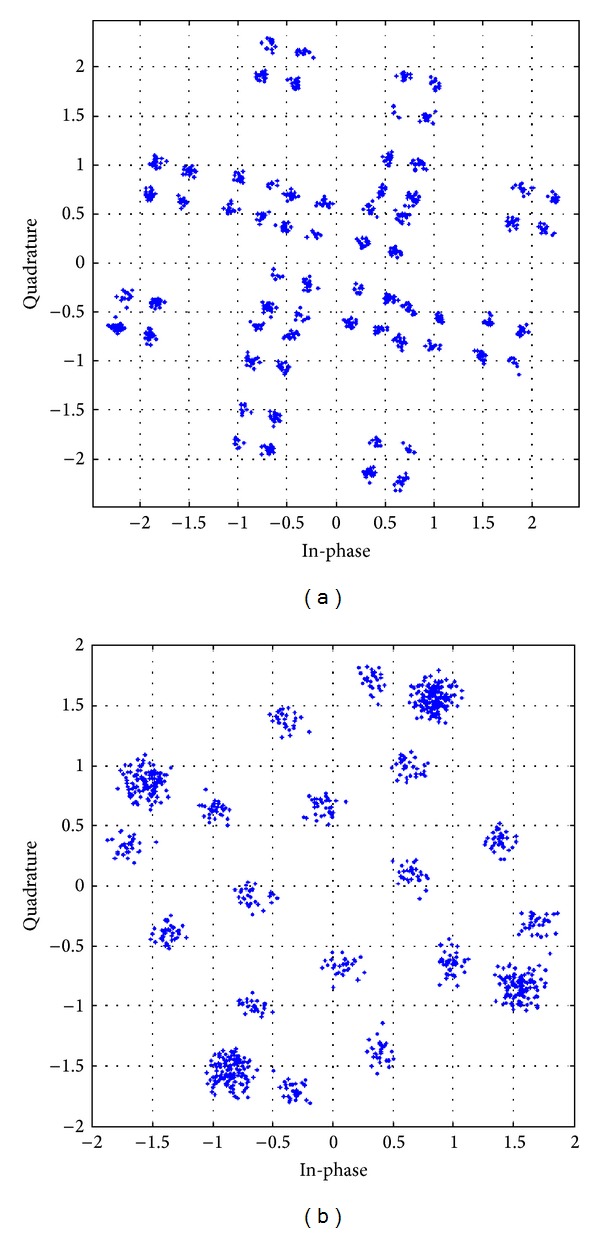
Received constellation for OSTBC MIMO antenna systems with code rate 2 with discriminating levels at SNR 40 dB and 30 dB for 3 × 3 and 4 × 4 antenna systems in (a) and (b), respectively, using QPSK modulation technique with angle 65.49° in (a) and 14.2° in (b).

The received constellation for different antenna configurations, with different modulation schemes utilizing the APASBCE technique, has been shown in further figures and their result analysis has been shown in Table 3. These received constellations show how much rotation is required for achieving the particular value of code rate, required for these STBC techniques to modulate through the channel using APASBCE scheme and to receive the ISI free symbols perfectively at the receiver. Figures 10(a) and 10(b) have been simulated for both the 16-QAM and QPSK whereas remaining figures from Figures 11–13 has been simulated for QPSK modulation only with their required rotation angle with different antenna configurations.

**Figure 14:**
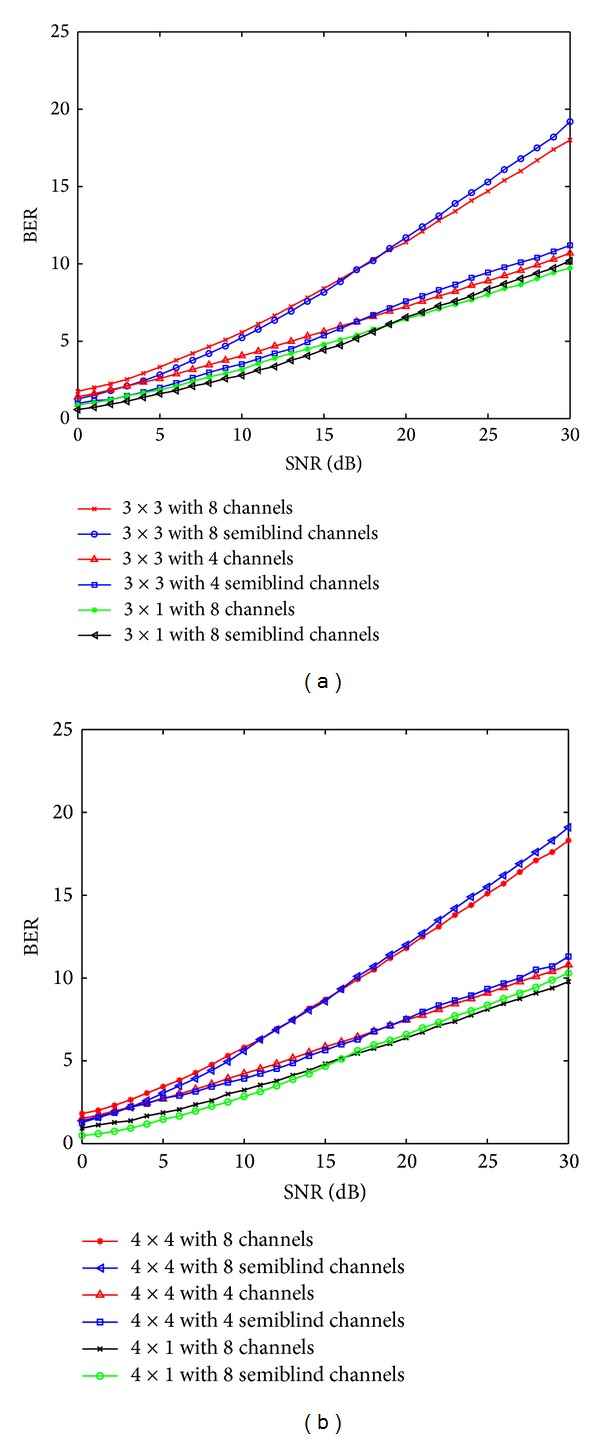
Capacity results for different antenna configurations with different channels.

It is also observable that the received signal improves with the increase in the number of antenna elements at the receiving end. We have used QPSK and 16-QAM modulation schemes using the gray constellation mapping for the comparative study of the existing results available in literature with the proposed APASBCE [18] scheme results for these mentioned modulation schemes. The capacity analysis and the improvement have already been discussed in [41] for the APASBCE based scheme using the existing MIMO systems available in the literature. Comparative study of the capacity improvement has been shown in Figure 14 using (48), (49), and (52) in [41], where the analysis has been done using 3 × 1, 3 × 3, 4 × 1, and 4 × 4 antenna systems for different channel numbers and obtained the improvement with the increase in the number of partial CSI channels.

Figures 14(a) and 14(b) show the improved results of APASBCE based capacity which shows that, for 3 × 1 antenna systems with 8 channels, the proposed system started to enhance the capacity at SNR level of 19.3 dB. Similarly for 3 × 3 antenna systems with 4 channels and 8 channels, it gave the improved enhancement of capacity at the SNR level of 17.9 dB, and 17.2 dB respectively. It also shows that the capacity improvement is related to the increase in the number of the channels. Again, for 4 × 1 antenna systems with 8 channels, the improvement started at the level of 16.4 dB, whereas for 4 × 4 antenna systems with 4 channels, it shows the improvement after 19.2 dB. For 4 × 4 antenna systems, the improvement in capacity has been found initially at the level of SNR 12.8 dB but then decreases and again improved after the level of 15.7 dB which then maintained its improvement after 20.6 dB. This variation was caused because of the fading effect and channel estimation adaptively using APASBCE method for stabilizing the channel state information.

Conclusively, this paper shows the comparison of the existing 16-QAM and QPSK modulation schemes for different code rates with the result of STBC with code rate higher than 1 using different STBC techniques with the new improved results found using the proposed APASBCE scheme, as discussed earlier, which shows the better BER result and improved capacity with less number of used training symbols and increasing the stability of the system by utilizing the minimum required number of training symbols. Also the constellation rotation for required angle has also been discussed for different higher code rate values for both 16-QAM and QPSK modulation schemes. These high code rate STBCs have been obtained and analyzed with improved results and the quantitative improvement has been discussed in this section.

## Figures and Tables

**Table 1:** Comparison of different STBCs for different transmitting antennas and code rates.

Design approach	Number of transmitting antennas	Number of symbols per block	Code rate	Complexity
Spatial multiplexing	*M*_*T*_	*M*_*T*_	*M*_*T*_	Less
Alamouti	2	2	1	Less
OSTBC	3, 4	3, 4	1/2	Medium
OSTBC	3, 4	3, 3	3/4	High
OSTBC	4	6, 8	1.3	Very high
OSTBC	3, 4	4	1.5	Very high
OSTBC	3, 4	4, 8	2	Very high
QOSTBC	4	4	1	High

**Table 2:** Comparison result analysis of different STBCs for different transmitting antennas with their code rates.

Modulation used	Antenna configuration	Code rate	STBC used	APASBCE based semiblind CE improvement	Remarks
QPSK	3 × 3	1/2	*S*_3_^*C*^ in (6)	After 17.4 dB SNR level	Alamouti's code comparison as shown in Figure 2.
16-QAM	3 × 3	1/2		After 12 dB SNR level	
QPSK	3 × 3	1/2	*S*_3_^*C*^ in (6) and *S*_3_^*C*^ in (7)	After 3.2 dB SNR level with BER 10^−4^ as compared with code rate 3/4	OSTBC comparison as shown in Figure 3.
16-QAM	3 × 3	1/2		After 2.4 dB SNR level with BER 10^−4^ as compared with code rate 3/4	
QPSK	4 × 4	1/2	*S*_4_^*C*^ in (6) and *S*_4_^*C*^ in (8)	After 1.6 dB SNR level with BER 10^−5^ as compared with code rate 3/4	OSTBC comparison as shown in Figure 4.
16-QAM	4 × 4	1/2		After 1.9 dB SNR level with BER 10^−5^ as compared with code rate 3/4	
QPSK	4 × 4	1.3	*A*′ and *B*′ in (44)	After 18.8 dB	OSTBC comparison as shown in Figure 5.
16-QAM	4 × 4	1	*S*_4_^*C*^ in (9)	After 14.2 dB SNR level before reaching the BER level of 10^−2^	QOSTBC and OSTBC comparison as shown in Figure 6.
QPSK	4 × 4	1.3	*A*′ and *B*′ in (44)	After 11.4 dB SNR level before reaching the BER level of 10^−2^	
QPSK	4 × 4	1.3 (8 Bps/Hz)	*A*′ and *B*′ in (44)	After 11.8 dB SNR level before reaching the BER of 10^−2^	OSTBC comparison as shown in Figure 7.
QPSK	4 × 4	1.3 (9 Bps/Hz)	*A*′ and *B*′ in (44)	After 16.7 dB SNR level before reaching the BER level of 10^−2^	
QPSK	4 × 4	2	*A*′ and *B*′ in (45)	After 16.2 dB	OSTBC comparison as shown in Figure 8.
16-QAM	4 × 4	2		After 20.8 dB	
QPSK	4 × 4	1.5	*A*′ and *B*′ in (47)	After 11.8 dB SNR level before reaching the BER level of 10^−3^	OSTBC comparison as shown in Figure 9.
16-QAM	4 × 4	1.5		After 18.9 dB SNR level before reaching the BER level of 10^−3^	

**Table 3:** Comparison of received constellation for different transmitting antennas with their code rates and required rotation angle.

Modulation type	Antenna configuration	Code rate	Discriminating SNR level	Required angle	Remarks
16-QAM	3 × 3	1	23 dB	36.89°	Figure 10(a)
QPSK	3 × 3	1	23 dB	8.92°	Figure 10(b)
QPSK	3 × 3	1/2	35 dB	1.5°	Figure 11(a)
QPSK	4 × 4	1/2	25 dB	18.48°	Figure 11(b)
QPSK	3 × 3	3/4	40 dB	1.8°	Figure 12(a)
QPSK	4 × 4	3/4	30 dB	3.2°	Figure 12(b)
QPSK	3 × 3	2	40 dB	65.49°	Figure 13(a)
QPSK	4 × 4	2	30 dB	14.2°	Figure 13(b)
